# First-line programmed death-1 inhibitor treatment for locoregionally advanced or metastatic cutaneous squamous cell carcinoma – A real-world experience from Israel

**DOI:** 10.3389/fonc.2023.1117804

**Published:** 2023-01-26

**Authors:** Itamar Averbuch, Saeed Salman, Noa Shtamper, Ilana Doweck, Aron Popovtzer, Gal Markel, Daniel Hendler, Inbar Finkel, Assaf Moore, Eyal Fenig, Tarek Taha, Kamel Mhameed, Noga Kurman, Salem Billan

**Affiliations:** ^1^ Davidoff Cancer Center, Rabin Medical Center – Beilinson Hospital, Petach Tikva Affiliated to Sackler Faculty of Medicine, Tel Aviv University, Tel Aviv, Israel; ^2^ The Joseph Fishman Oncology Center, Rambam Health Care Campus, Haifa, Affiliated to the Rappaport Faculty of Medicine, Israel Institute of Technology-Technion, Haifa, Israel; ^3^ Rappaport Faculty of Medicine, Technion – Israel Institute of Technology, Haifa, Israel; ^4^ Department of Otolaryngology-Head and Neck Surgery, Carmel Medical Center, Haifa, Affiliated to the Rappaport Faculty of Medicine, Israel Institute of Technology-Technion, Haifa, Israel; ^5^ Sharett Institute of Oncology, Hadassah Medical Center, Jerusalem, Affiliated with the Hebrew University of Jerusalem, Jerusalem, Israel; ^6^ Oncology Institute, Baruch Padeh Medical Center, Poriya, Affiliated with Azrieli Faculty of Medicine, Bar Ilan University, Poriya, Israel

**Keywords:** PD-1 inhibitor, cemiplimab, pembrolizumab, radiotherapy, cutaneous squamous cell carcinoma (cSCC), real-world experience

## Abstract

**Objective:**

Cutaneous squamous cell carcinoma (cSCC) is the second most common non-melanoma skin cancer worldwide. It is usually treated surgically, with very high cure rates. However, in 3%-7% of cases, cSCC metastasizes to lymph nodes or distant organs. Many of the affected patients are elderly with comorbidities who are not candidates for standard-of-care curative-intent treatment with surgery and/or radio-/chemotherapy. Immune checkpoint inhibitors, which target programmed cell death protein 1 (PD-1) pathways, have recently emerged as a potent therapeutic option. The present report presents the Israeli experience with PD-1 inhibitors for the treatment of loco-regionally advanced or metastatic cSCC in a diverse and elderly population, with or without the addition of radiotherapy.

**Material and methods:**

The databases of two university medical centers were retrospectively searched for patients with cSCC treated with the PD-1 inhibitors cemiplimab or pembrolizumab between January 2019 and May 2022. Data on baseline, disease-related, treatment-related, and outcome parameters were collected and analyzed.

**Results:**

The cohort included 102 patients of a median age 78.5 years. Evaluable response data were available for 93. The overall response rate was 80.6%: complete response in 42 patients (45.2%) and partial response in 33 (35.5%). Stable disease was recorded in 7 (7.5%) and progressive disease in 11 (11.8%). Median progression-free survival was 29.5 months. Radiotherapy was administered to the target lesion during PD-1 treatment in 22.5% of patients. mPFS was not significantly different in patients who treated with RT than patients how did not (NR vs 18.4 months, HR=0.93, 95%CI: 0.39 - 2.17, p<0.859). Any-grade toxicity was recorded in 57 patients (55%), including grade ‗3 in 25, of whom 5 (5% of cohort) died. Compared to toxicity-free patients, patients with drug toxicity had better progression-free survival (18.4 months vs not reached, HR=0.33, 95% CI: 0.13-0.82, p=0.012) and higher overall response rate (87% vs 71.8%, p=0.06).

**Conclusion:**

This retrospective real-world study showed that PD-1 inhibitors were effective in the treatment of locally advanced or metastatic cSCC and appeared to be amenable for use in elderly or fragile patients with comorbidities. However, the high toxicity warrants consideration against other modalities. Induction or consolidation radiotherapy may improve the results. These findings need to be corroborated in a prospective trial.

## Introduction

Cutaneous squamous cell carcinoma (cSCC) is the second most common skin cancer, exceeded only by BCC in incidence (1, 2). Risk factors for cSCC include, ultraviolet radiation exposure, advanced age, immunosuppression, chronic inflammation and non-healing wounds, smoking, and various genetic disorders such as genodermatosis (3–6).

Current standard of care treatment for local disease is surgical resection, followed when indicated by adjuvant radiotherapy. However, the most recent revised NCCN guidelines suggest that definitive radiotherapy can be pursued in non-operable cases: when surgery might result in significant cosmetic or functional morbidity or by patient’s preference (7).

More than 95% of patients with early-stage cSCC are cured with surgery (8). In a retrospective study of 598 patients with cSCC treated with local excision as a single modality, only 6.7% had disease recurrence (local or lymphatic spread) (9). However, in 3%-7% of patients, the disease metastasizes to regional lymph nodes or distant organs (9–11).

Immune checkpoint inhibitors (ICIs) are monoclonal antibodies that target the programmed cell death protein 1 (PD-1) pathways to enhance the immune response. For patients with advanced cSCC who are not amenable to curative-intent treatment with surgery and/or chemo-/radiotherapy, high-affinity PD-1 inhibitors such as cemiplimab or pembrolizumab are emerging as a viable option for first-line treatment. Phase II cohort studies have shown objective response rates of 35-60% in patients with nonoperable or metastatic disease (12–15). Cemiplimab may also serve as neoadjuvant therapy for locally advanced stage II-IV(M0) cSCC. A recent phase II study reported a 51% complete pathological response rate and a further 13% major pathological response rate after up to four doses of 350 mg every 3 weeks (16).

Locally advanced cSCC is more common in the elderly (17) and in populations with severe comorbidities (18, 19) who are not eligible for radical surgery or curative-intent radiotherapy and require upfront systemic therapy. However, data on the management of this patient group remain sparse because their fragile state excludes them from participation in clinical trials. Therefore, real-world studies are crucial. The aim of the present report was to describe the experience of two university medical centers with PD-1 inhibitor in the treatment of a diverse and elderly population with locally advanced or metastatic cSCC. Because of the complexity of the treatment decisions, patients with borderline operable disease were included in the cohort.

## Methods

### Patients

The study population consisted of all patients treated for cSCC with cemiplimab or pembrolizumab at the Davidoff Cancer Center in Rabin Medical Center and the Fishman Oncology Center in Rambam Medical Center between January 2019 and May 2022.

### Clinical data

Data were collected retrospectively from the electronic medical records, as follows: demographic details, tumor histology, disease stage at presentation, and course of treatment. The overall response rate (ORR) to PD-1 inhibitor treatment was recorded in addition to the progression-free survival (PFS) and treatment-related adverse events. Response was categorized as complete response (CR), partial response (PR), stable disease (SD), and progressive disease (PD). ORR was defined as the percentage of patients who achieved a CR or PR. Response characterization was performed by the treating oncologist using the immune-related Response Evaluation Criteria in Solid Tumors (iRECIST) criteria. Clinical PR or CR (cPR or cCR) was determined by the physical examination of the attending oncologist The safety profile was defined as the incidence of treatment-related adverse events according to the Common Terminology Criteria for Adverse Events (CTCAE) version 5.0.

### Statistical analysis

All statistical analyses were performed using SPSS software (IBM Corp.), version 25. Chi-square of independence test and log rank test were performed to examine the relationship of cognitive status, immune status, and toxicity with different aspects of response to PD-1 inhibitors. Reverse Kaplan-Meier method was used to calculate follow-up time. Kaplan-Meier method was used to evaluate PFS. P values <0.05 were considered significant. The study was approved by the local Research Ethics Committees.

## Results

The cohort included 102 patients diagnosed with locally advanced or metastatic cSCC. 78.4% men and 21.6% women; 83.1% were of Jewish Ashkenazi origin and the remainder were divided between Jews of North African descent and Arabs (6.5% each). At the time of PD-1 inhibitor treatment, 45 patients had stage IV disease, 49 stage III, 4 stage II, and 2 stage I (located in the eyelid skin in both); data on disease stage were missing for 2 patients. The most common primary tumor site was the head and neck, in 69% of cases, followed by various other body locations in 14%. In 18% of cases, disease presentation was local lymphatic or metastatic spread alone, without a distinct primary lesion identified (unknown primary). Treatment consisted of cemiplimab in 100 patients and pembrolizumab in 2. Median age at onset of PD-1 inhibitor treatment was 78.5 years (range 51-96); 13 patients (13%) where older than 90 years. Additional baseline characteristics are listed in **Table 1**.

**Table 1:** Clinical characteristics of patients with cSCC.

Characteristics	Value
Age, years (median) (n=102)	78.5
Sex (n=102)	
Male	80 (78.4%)
Female	22 (21.6%)
Cognitive status (n=101)	
Cognitively preserved	91 (90.1%)
Mild cognitive impairment	8 (7.9%)
Dementia	2 (2%)
Immune suppression (n=102)	
Without immunosuppression	87 (85.3%)
Organ transplant	5 (4.9%)
Hematologic malignancy	6 (5.9%)
Rheumatologic drugs	3 (2.9%)
Other	1 (1%)
Primary location(n-102)	
Face, T-zone (forehead & nose)	19 (18.6%)
Face, other	24 (23.5%)
Upper limb/trunk	13 (12.7%)
Ear	9 (8.8%)
Lower limb	1 (1%)
Scalp	18 (17.7%)
Unknown primary	18 (17.7%)
Treatment-naive at ICI tx (n=93)	
Yes	31 (33.3%)
No	62 (66.7%)
Origin (n=77)	
North African Jews	5 (6.5%)
European Jews	64 (83.1%)
Arabs	5 (6.5%)
Others	3 (3.9%)

Values given as n(%) unless otherwise indicated.

cSCC, cutaneous squamous cell carcinoma; ICI, immune checkpoint inhibitor.

The median duration of follow-up from the time of cSCC diagnosis (including early-stage disease) was 28.33 months, and from onset of PD-1 inhibitor treatment, 14.6 months. Nine patients died before a response assessment could be made. Two of them died of ICI-related toxicity, including one case of myocarditis and one case of Guillain-Barré syndrome. Four patients died of other comorbidities unrelated to ICI, and 3 patients died of unknown causes.

Among the 93 patients in whom a response assessment was available, the overall response rate (ORR) was 80.6%. The best response was CR in 42 patients (45.2%), including the 2 patients treated with pembrolizumab, PR in 33 (35.5%), SD in 7 (7.5%), and PD in 11 (11.8%). The median progression-free survival (PFS) was 29.5 months. The median PFS was significantly better in patients who achieved a CR than in patients who achieve a PR or SD (29.5 vs. 16.9 months, HR=0.045, 95%CI: 0.006 -0.373, p<0.001). The median time to cPR was 1.6 months (range, 0.23–8.5 months), and to cCR, 3.45 months (range, 0.6-15.9 months). At data cutoff, 63 patients (84% of responders) continued to respond. Radiotherapy was administered to the target lesion during PD-1 inhibitor treatment in 22.5% of patients; in 4 cases as induction treatment at initiation of PD-1 inhibitors, in 9 cases as consolidation at maximal response, in 6 cases as salvage at progression, and in 2 cases as palliation. Median PFS was not significantly different in patients who received RT compared with patients who did not (NR vs 18.4 months, HR=0.93, 95%CI: 0.39 - 2.17, p<0.859). RT was given with IMRT-based planning in hypofractionated or SBRT doses.

PD-1 inhibitors were the first treatment modality in 31 of the 102 patients (30%). Within this subgroup, ORR was 80.0%, with 12 patients (38.7%) achieving CR, 13 (41.9%) PR, 2 patients (6.5%) had SD, and 4 (12.9%) had PD. There was no statistically significant difference in PFS between patients who received PD-1 inhibitors as 1-line and those who received it as salvage after progression or recurrence (29.5 vs NR, HR=1.1, 95%CI: 0.41 - 2.75 p=0.91). At the time of data cutoff, 20 patients (80% of first-time responders) continued to respond.

Fifteen patients in the cohort (14.7%) were immune-compromised due to solid organ (kidney) transplant (n=5), hematological malignancy (n=7), and immunomodulatory treatment received for rheumatological disease (n=3). Three of them died before a response assessment could be made. Among the transplant recipients, there was not a single rejection of the transplanted organ. There was no difference between the immune-compromised and immune-competent patients in ORR (81.8% vs 80.5%, p=0.916), median PFS (18.4 vs. 29.5 months, HR=1.64, 95% CI 0.38 -7.04, p=0.51) or any-grade toxicity (53.5% vs 56.3%, p=0.83).

**Figure 1:**
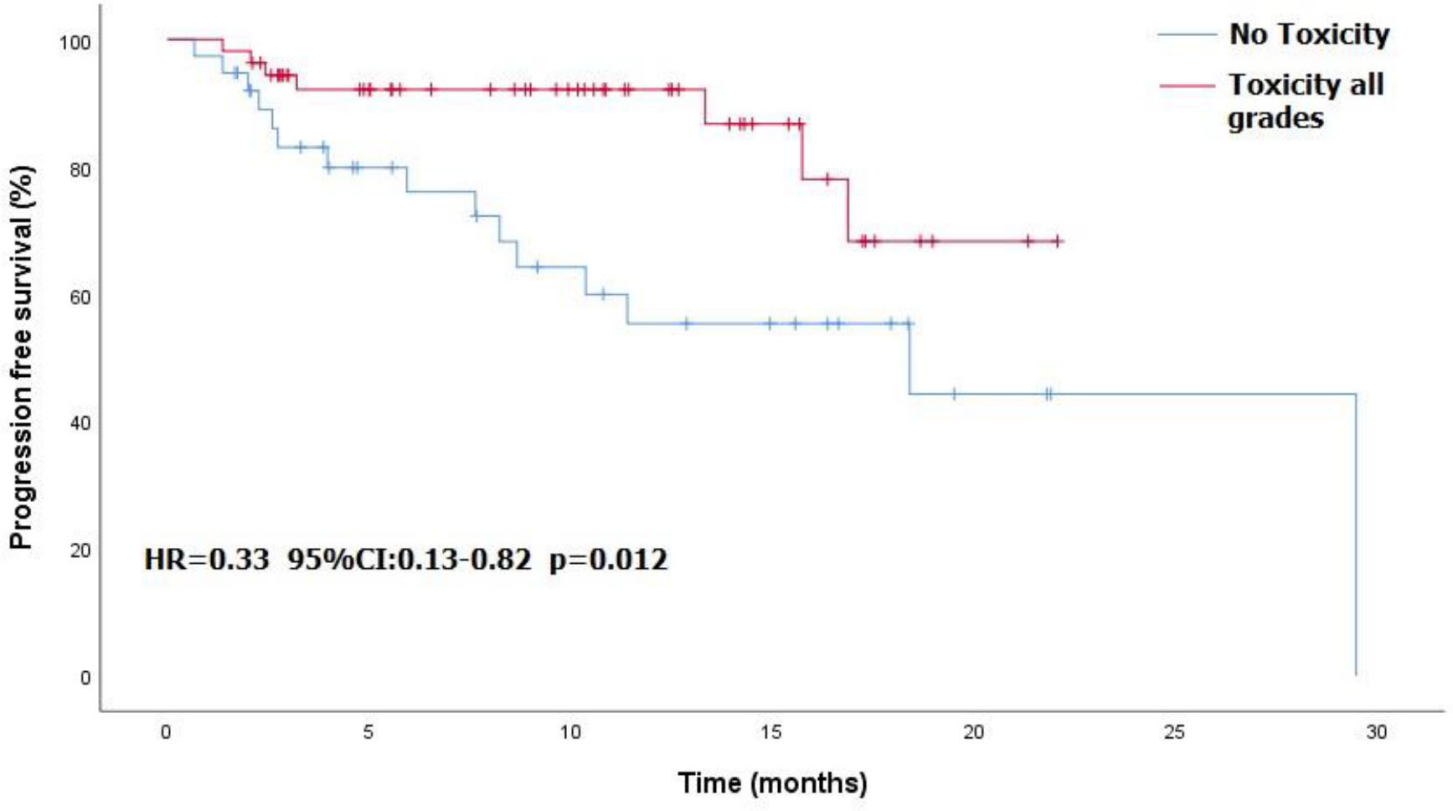
Progression-free survival in patients with and without toxicity.

Overall, 57 patients (55%) experienced treatment-related toxicity, including 25 (24%) with grade 3 or more. Five patients died of toxicity: 2 pneumonitis, 1 encephalitis, 1 myocarditis, and 1 Guillain-Barré syndrome. **Table 2** presents the demographic characteristics of these patients. Grade 3-4 toxicities included dermatitis in 7 patients, fatigue in 6, colitis in 3, arthralgia in 2, and pneumonitis, Raynaud syndrome, thyroiditis, Guillain-Barré syndrome, myasthenia gravis, and hearing loss in 1 patient each. **Table 3** details the full list and toxicity grading. There was no correlation between risk of toxicity and patients’ age, gender, origin, immune status, or cognitive status. PFS was significantly better in patients who experienced any type of toxicity than in patients with no toxicity (18.4 months vs. NR, HR=0.33, 95%CI: 0.13 -0.82, p=0.012). The Kaplan-Meier curve is presented in **Figure 1**. A similar albeit nonsignificant trend was found for ORR (87% vs 71.8%, respectively, p=0.06).

**Table 2:** Demographic characteristics of patients developed grade 5 toxicity.

	Gender	Medical history	Age	Cause of death
1	Male	COPD, AAA	84	Pneumonitis
2	Male	AF, IHD HTN, PV, MCI	85	Gulian bare
3	Female	TAVI, DM2, HTN, Dyslipidaemia, AF	87	Myocarditis
4	Male	Dyslipidaemia, HTN, CRF, Gout	75	Encephalitis
5	Male	Epilepsy, s/p CVA, HTN	72	Pneumonitis

AAA, Abdominal aortic aneurysm; PV, polycythemia vera; HTN, Hypertention; CRF, Chronic renal failure; COPD, chronic obstructive pulmonary disease; AF, Atrial fibrilation; MCI, mild cognitive impairment; TAVI, Transcatheter Aortic-Valve Implantation.

**Table 3:** Adverse event profile of 102 ICI-treated patients with cSCC.

	Grade 1-2 (%)	Grade 3-4 (%)	Grade 5 (%)	Total (%)
Ageusia	7 (6.8%)	0 (0%)	0 (0%)	7 (6.8%)
Anemia	6 (5.8%)	0 (0%)	0 (0%)	6 (5.8%)
Arthralgia	6 (5.8%)	2 (1.9%)	0 (0%)	8 (7.8%)
Asthenia	2 (1.9%)	0 (0%)	0 (0%)	2 (1.9%)
Colitis	5 (4.9%)	3 (2.9%)	0 (0%)	8 (7.8%)
Cytopenia	1 (0.9%)	0 (0%)	0 (0%)	1 (0.9%)
Dermatitis	14 (13.7%)	7 (6.8%)	0 (0%)	21 (20.5%)
Encephalitis	0 (0%)	0 (0%)	1 (1%)	1 (0.9%)
Fatigue	13 (12.7%)	6 (5.8%)	0 (0%)	19 (18.6%)
Guillain-Barré	0 (0%)	1 (0.9%)	1 (1%)	2 (1.9%)
Hepatitis	1 (0.9%)	1 (0.9%)	0 (0%)	2 (1.9%)
Myasthenia gravis	0 (0%)	1 (0.9%)	0 (0%)	1 (0.9%)
Myocarditis	0 (0%)	1 (0.9%)	1 (1%)	2 (1.9%)
Nausea	2 (1.9%)	0 (0%)	0 (0%)	2 (1.9%)
Ototoxicity	0 (0%)	1 (0.9%)	0 (0%)	1 (0.9%)
Pneumonitis	1 (0.9%)	1 (0.9%)	2 (2%)	4 (3.9%)
Psoriasis	1 (0.9%)	0 (0%)	0 (0%)	1 (0.9%)
Raynaud’s syndrome	0 (0%)	1 (0.9%)	0 (0%)	1 (0.9%)
Thyroiditis	5 (4.9%)	1 (0.9%)	0 (0%)	6 (5.8%)
Weight lose	5 (4.9%)	0 (0%)	0 (0%)	5 (4.9%)

Values given as n(%).

ICI, immune checkpoint inhibitor; cSCC, cutaneous squamous cell carcinoma.

## Discussion

The present study describes the real-world Israeli experience with PD-1 inhibitors for the treatment of cSCC. The results revealed a high response rate and prolonged progression-free survival. All patients were treated in two tertiary academic facilities by trained oncologists as part of a disease management team.

Special attention was addressed to patients with previously untreated disease. In the clinical trial setting, the only available data pertaining to this population may be derived from the CARSKIN study on pembrolizumab effectiveness in 57 patients (15). The ORR was 42%, considerably lower than the 80% in our cohort wherein 39% achieved CR and PFS was 29.5 months. This discrepancy may be explained by the utilization of radiotherapy in 22.5% of our patients. Previous publications have also shown an association between induction or consolidation RT and improved outcomes (20, 21). The mPFS of patients who received RT was not significantly different from the mPFS of the rest of the population. According to the indications for RT, one can assume that this population had a more aggressive disease course and shorter PFS. In fact, this the lack of a significant difference may indicate that RT renders their outcomes comparable to the general population’s.

Although the standard of care for newly diagnosed resectable cSCC is surgery, recent clinical trials have shown that PD-1 inhibitors may significantly contribute to treatment. Gross et al. (16) found that in the neoadjuvant setting, four cycles of cemiplimab yielded a 51% pathological CR. Together with the present study, these findings may imply that conservative treatment with ICIs and induction/consolidative radiotherapy is a viable surgery-sparing option for carefully selected patients. Notably, the time to response may be especially long with PD-1 inhibitors, reaching about 8 months in some cases; therefore, treatment continuation should be considered even in patients initially deemed unresponsive by the iRECIST criteria.

Overall, 57 patients (55%) in our cohort experienced some grade of toxicity; 25 (24%) with grade 3 or more, of whom 5 (4.9% of total cohort) died. In the clinical trial setting, the KEYNOTE-629 study reported an 11.9% rate of adverse events related to grade 3-5 treatment-induced toxicity (10), with 2 related deaths (1.25%) (14), and the CARSKIN trial reported 4 patients (7%) with grade 3 or more toxicity and 1 related (2.56%) death (15). In the cemiplimab trial, Migden et al. (9) documented treatment-emergent adverse events in 34 of 78 patients (44%) with grade 3-4 toxicity, among whom 1 (1.28%) died (13). Patients in the present cohort who experienced some grade of toxicity showed a better response to treatment, in terms of both response rate and PFS, than patients without toxicity. This phenomenon has been observed in a number of malignancies and apparently represents a bystander effect from activated immune cells (22).

Real-world data are especially important in cSCC because of the baseline characteristics of this population. Patients with cSCC tend to be older with a broad range of co-morbidities that could exclude them from recruitment to clinical trials (e.g. ECOG 3-4, solid organ transplant, immunosuppressive therapy, hematologic malignancies) (23–25). Therefore, data are lacking for a considerable proportion of the treatment population. Our study showed that various types of immunosuppression did not affect the effectiveness of anti-PD-1 treatment, supporting previous research (23). The ORR in the real world was significantly higher than that in clinical studies, but the toxicity profile was much more severe, with more than double the grade 5 toxicity rate. As can be seen in **Table 2**, the grade 5 toxicity rate demonstrated in this study may be explained by the fragility of this group. As no correlation was found between toxicity and different baseline characteristics, we suggest that treatment be given with caution and under close supervision with aggressive toxicity management.

This study’s main limitations are related to its retrospective designand its inherent biases, including the difficulty in drawing conclusions about causality. Another limitation is, the uncontrolled sample size and the, heterogeneous nature of the population. In addition, this is a study from only two medical institutions in the same country.

## Conclusion

This retrospective study showed that PD-1 inhibitors appear to be a suitable option for the treatment of patients with locally advanced or metastatic cSCC, This applies also to elderly and immune-suppressed patients, albeit with consideration of the high drug toxicity. Induction or consolidation radiotherapy may improve the results. These findings need to be corroborated in a prospective trial.

## Data availability statement

The raw data supporting the conclusions of this article will be made available by the authors, without undue reservation.

## Ethics statement

The studies involving human participants were reviewed and approved by the Helsinki Committee of Rabin medical center, Petch-Tikva, Israel, and Rambam Health Care Campus, Haifa, Israel. Written informed consent for participation was not required for this study in accordance with the national legislation and the institutional requirements.

## Author contributions

IA, SS, NK, and SB took the lead in writing the manuscript. All authors contributed to the article and approved the submitted version.

## References

[1] Rogers HW Weinstock MA Feldman SR Coldiron BM. Incidence estimate of nonmelanoma skin cancer (Keratinocyte carcinomas) in the US population, 2012. JAMA Dermatol (2015) 151(10):1081–6. 10.1001/jamadermatol.2015.1187 25928283

[2] Lomas A Leonardi-Bee J Bath-Hextall F. A systematic review of worldwide incidence of non-melanoma skin cancer. Br J Dermatol (2012) 166(5):1069–80. doi: 10.1111/j.1365-2133.2012.10830.x 22251204

[3] Stratigos A Garbe C Lebbe C Malvehy J Del Marmol V Pehamberger H. Diagnosis and treatment of invasive squamous cell carcinoma of the skin: European consensus-based interdisciplinary guideline. Eur J Cancer (2015) 51(14):1989–2007. 26219687 10.1016/j.ejca.2015.06.110

[4] Gül Ü Kiliç A. Squamous cell carcinoma developing on burn scar. Ann Plast Surg (2006) 56(4):406–8. 10.1097/01.sap.0000200734.74303.d5 16557073

[5] Leonardi-Bee J Ellison T Bath-Hextall F. Smoking and the risk of non-melanoma skin cancer: Systematic review and meta-analysis. Arch Dermatol (2012) 148(8):939–46. 10.1001/archdermatol.2012.1374 22711192

[6] Fine JD Johnson LB Weiner M Li KP Suchindran C. Epidermolysis bullosa and the risk of life-threatening cancers: The national EB registry experience, 1986-2006. J Am Acad Dermatol (2009) 60(2):203–11. 10.1016/j.jaad.2008.09.035 19026465

[7] Schmults CD Blitzblau R Aasi SZ Alam M Andersen JS Baumann BC. NCCN guidelines® insights: Squamous cell skin cancer, version 1.2022. J Natl Compr Canc Netw (2021) 19(12):1382–94. 10.6004/jnccn.2021.0059 34902824

[8] Arielle AN Arpey CJ Hruza G Olbricht SM Bennett R. Consensus for nonmelanoma skin cancer treatment, part II: Squamous cell carcinoma, including a cost analysis of treatment methods. Dermatologic Surg (2015) 41(11):1214–40. 10.1097/DSS.0000000000000478 26445288

[9] Khan K Mykula R Kerstein R Rabey N Bragg T Crick A. A 5-year follow-up study of 633 cutaneous SCC excisions: Rates of local recurrence and lymph node metastasis. J Plast Reconstr Aesthetic Surg (2018) 71(8):1153–8. doi: 10.1016/j.bjps.2018.03.019 29803777

[10] Genders RE Osinga JAJ Tromp EE O’Rourke P Bouwes Bavinck JN Plasmeijer EI. Metastasis risk of cutaneous squamous cell carcinoma in organ transplant recipients and immunocompetent patients. Acta Derm Venereol (2018) 98(6):551–5. 10.2340/00015555-2901 29405246

[11] Karia PS Han J Schmults CD. Cutaneous squamous cell carcinoma: Estimated incidence of disease, nodal metastasis, and deaths from disease in the united states, 2012. J Am Acad Dermatol (2013) 68(6):957–66. doi: 10.1016/j.jaad.2012.11.037 23375456

[12] Migden MR Rischin D Schmults CD Guminski A Hauschild A Lewis KD. PD-1 blockade with cemiplimab in advanced cutaneous squamous-cell carcinoma. N Engl J Med (2018) 379(4):341–51. doi: 10.1056/NEJMoa1805131 29863979

[13] Migden MR Khushalani NI Chang ALS Lewis KD Schmults CD Hernandez-Aya L. Cemiplimab in locally advanced cutaneous squamous cell carcinoma: results from an open-label, phase 2, single-arm trial. Lancet Oncol (2020) 21(2):294–305. 31952975 10.1016/S1470-2045(19)30728-4 PMC7771329

[14] Hughes BGM Munoz-Couselo E Mortier L Bratland Gutzmer R Roshdy O. Pembrolizumab for locally advanced and recurrent/metastatic cutaneous squamous cell carcinoma (KEYNOTE-629 study): An open-label, nonrandomized, multicenter, phase II trial. Ann Oncol (2021) 32(10):1276–85. 10.1016/j.annonc.2021.07.008 34293460

[15] Maubec E Boubaya M Petrow P Beylot-Barry M Basset-Seguin N Deschamps L. Phase II study of pembrolizumab as first-line, single-drug therapy for patients with unresectable cutaneous squamous cell carcinomas. J Clin Oncol (2020) 38(26):3051–61. 10.1200/JCO.19.03357 32730186

[16] Gross ND Miller DM Khushalani NI Divi V Ruiz ES Lipson EJ. Neoadjuvant cemiplimab for stage II to IV cutaneous squamous-cell carcinoma. N Engl J Med (2022) 387(17):1557–68. doi: 10.1056/NEJMoa2209813 PMC9844515 36094839

[17] Gray DT. Trends in the population-based incidence of squamous cell carcinoma of the skin first diagnosed between 1984 and 1992. Arch Dermatol (1997) 133(6):735. 9197827

[18] Omland SH Ahlström MG Gerstoft J Pedersen G Mohey R Pedersen C. Risk of skin cancer in patients with HIV: A Danish nationwide cohort study. J Am Acad Dermatol (2018) 79(4):689–95. 10.1016/j.jaad.2018.03.024 29588249

[19] Berg D Otley CC. Skin cancer in organ transplant recipients: Epidemiology, pathogenesis, and management. J Am Acad Dermatol (2002) 47(1):1–20. 12077575 10.1067/mjd.2002.125579

[20] van Hagen P Hulshof MCCM van Lanschot JJB Steyerberg EW Henegouwen MI van B Wijnhoven BPL. Preoperative chemoradiotherapy for esophageal or junctional cancer. N Engl J Med (2012) 366(22):2074–84. doi: 10.1056/nejmoa1112088 22646630

[21] Palma DA Olson R Harrow S Gaede S Louie AV Haasbeek C. Stereotactic ablative radiotherapy versus standard of care palliative treatment in patients with oligometastatic cancers (SABR-COMET): A randomised, phase 2, open-label trial. Lancet (2019) 393(10185):2051–8. 10.1016/S0140-6736(18)32487-5 30982687

[22] Das S Johnson DB. Immune-related adverse events and anti-tumor efficacy of immune checkpoint inhibitors. J Immunother Cancer (2019) 7(1):1–11. doi: 10.1186/s40425-019-0805-8 31730012 PMC6858629

[23] Leiter U Loquai C Reinhardt L Rafei-Shamsabadi D Gutzmer R Kaehler K. Original research: Immune checkpoint inhibition therapy for advanced skin cancer in patients with concomitant hematological malignancy: a retrospective multicenter DeCOG study of 84 patients. J Immunother Cancer (2020) 8(2). 10.1136/jitc-2020-000897 PMC7583786 33093156

[24] Chapman JR Webster AC Wong G. Cancer in the transplant recipient . Available at: http://perspectivesinmedicine.cshlp.org/. 10.1101/cshperspect.a015677 PMC3685882 23818517

[25] Grulich AE Van Leeuwen MT Falster MO Vajdic CM. Incidence of cancers in people with HIV/AIDS compared with immunosuppressed transplant recipients: a meta-analysis. Lancet (2007) 370:59–67. 10.1016/S0140-6736(07)61050-2 17617273

